# On Spasmodic Asthma

**Published:** 1853

**Authors:** Eben Watson


*On Spasmodic Asthma.*—By Eben Watson, M. D.

Dr. Eben Watson sums up his views upon the nature and treatment of asthma in the following manner:—

1st. That very many cases of bronchial asthma have their origin in laryngeal disease; that some remain for a variable period, as a spasmodic affection of the glottidean muscles, and that in all cases of the disease in question, although the bronchi have long been affected, the chief contraction still occurs in the larynx.

2d. That if this contraction at the glottis be in any way overcome, that of the smaller bronchi either simultaneously or speedily relaxes.

3d. That the usual remedies employed in cases of spasmodic asthma, are either such as are directed against the complications of the disease, and not against its proximate cause, or such as have been found in practice incapable of accomplishing its removal. The latter are therefore useless, and the former unfit to fulfil the indication referred to above.

4th. But this indication may be answered more or less perfectly in different cases, by the application of a solution of caustic of moderate strength (grs. xv., or a qr. to half oz.) to the glottis, which is the organ chiefly affected.

5th. Cardiac asthma, as it is called, does not usually depend proximately on simple spasmodic contraction of the bronchial tubes, but rather on vesicular emphysema. Cases of this kind are therefore unfit for topical treatment.

6th. Electricity passed in gentle currents, as much as possible along the bronchial tubes, may be found to diminish their contractility; and repeated small doses of strychnia may likewise co-operate with the other means of treatment, probably by withdrawing the nervous energy to other parts, at a distance from the affected air-tubes.

***Glasgow Medical Journal,*** April, 1853.

